# pH-Driven Intracellular Nano-to-Molecular Disassembly of Heterometallic [Au_2_L_2_]{Re_6_Q_8_} Colloids (L = PNNP Ligand; Q = S^2−^ or Se^2−^)

**DOI:** 10.3390/nano12183229

**Published:** 2022-09-17

**Authors:** Bulat Faizullin, Irina Dayanova, Igor Strelnik, Kirill Kholin, Irek Nizameev, Aidar Gubaidullin, Alexandra Voloshina, Tatiana Gerasimova, Ilya Kashnik, Konstantin Brylev, Guzel Sibgatullina, Dmitry Samigullin, Konstantin Petrov, Elvira Musina, Andrey Karasik, Asiya Mustafina

**Affiliations:** 1 Arbuzov Institute of Organic and Physical Chemistry, FRC Kazan Scientific Center of RAS, 8 Arbuzov Street, 420088 Kazan, Russia; 2 Department of Nanotechnology in Electronics, Kazan National Research Technical University Named after A.N. Tupolev-KAI, 10 K. Marx Street, 420111 Kazan, Russia; 3 Nikolaev Institute of Inorganic Chemistry, SB RAS, 3 Academician Lavrentiev Avenue, 630090 Novosibirsk, Russia; 4 Kazan Institute of Biochemistry and Biophysics, FRC Kazan Scientific Center of RAS, 2/31 Lobachevski Street, 420111 Kazan, Russia; 5 Institute for Radio-Electronics and Telecommunications, Kazan National Research Technical University Named after A.N. Tupolev-KAI, 10 K. Marx Street, 420111 Kazan, Russia

**Keywords:** hexarhenium clusters, Au(I) complex, pH-triggered disassembly, rapture of lysosomal membrane, apoptotic pathway

## Abstract

The present work introduces a simple, electrostatically driven approach to engineered nanomaterial built from the highly cytotoxic [Au_2_L_2_]^2+^ complex (Au_2_, L = 1,5-bis(p-tolyl)−3,7-bis(pyridine-2-yl)−1,5-diaza-3,7-diphosphacyclooctane (PNNP) ligand) and the pH-sensitive red-emitting [{Re_6_Q_8_}(OH)_6_]^4−^ (Re_6_-Q, Q = S^2−^ or Se^2−^) cluster units. The protonation/deprotonation of the Re_6_-Q unit is a prerequisite for the pH-triggered assembly of Au_2_ and Re_6_-Q into Au_2_Re_6_-Q colloids, exhibiting disassembly in acidic (pH = 4.5) conditions modeling a lysosomal environment. The counter-ion effect of polyethylenimine causes the release of Re_6_-Q units from the colloids, while the binding with lysozyme restricts their protonation in acidified conditions. The enhanced luminescence response of Re_6_-S on the disassembly of Au_2_Re_6_-S colloids in the lysosomal environment allows us to determine their high lysosomal localization extent through the colocalization assay, while the low luminescence of Re_6_-Se units in the same conditions allows us to reveal the rapture of the lysosomal membrane through the use of the Acridine Orange assay. The lysosomal pathway of the colloids, followed by their endo/lysosomal escape, correlates with their cytotoxicity being on the same level as that of Au_2_ complexes, but the contribution of the apoptotic pathway differentiates the cytotoxic effect of the colloids from that of the Au_2_ complex arisen from the necrotic processes.

## 1. Introduction

The wide and personalized differences between tumors and their ability to evolve over time or acquire multidrug resistance (MDR) warrant a diverse plethora of treatment options. The treatment of drug-resistant cancer is a challenging problem for which alternative approaches to chemotherapy are being developed. In this regard, it is worth noting the silencing of the expression of tumor-associated genes as an alternative or complementary route to the use of chemotherapeutic agents [1,2,3,4]. However, the present work is devoted to nanomaterial’s engineering strategy aimed at damaging specific cellular compartments as an alternative approach to overcoming drug resistance. Lysosomes are biorelevant cell compartments that are well-known to be overdeveloped in cancer versus normal cells [5,6]. Damage to lysosomes due to lysosome membrane permeabilization (LMP) can derive from swelling [7], agglomeration [8,9], or the dissolution of a nanomaterial within lysosomes [10,11,12,13,14,15,16,17], in turn resulting from specific lysosomal acidity (pH < 5.0). The aforesaid behavior of the nanomaterial can be achieved through developing specific nanoarchitectures exhibiting either pH-dependent aggregation or dissolution behavior and high affinity to lysosomal compartments. The literature data represent excellent examples of the nanoarchitectures generating specific cancer versus normal cell death [7,8,9,10,11,12,13,14,15,16,17], although the main structural or morphological features required for such specificity are not well recognized.

The artificial inorganic nanoparticles built from metal oxides, sulfides, and noble metals designated as engineered nanomaterials (ENs) have gained growing attention as a promising basis for anticancer therapeutic agents [18,19]. The bottom-up approach in the development of ENs through the use of metal complexes as building blocks in developing either crystalline or gel-like nanomaterial is gaining growing attention since the approach can provide an additional tool to develop anticancer treatment through the controlled release of metal complexes instead of metal ions in the lysosomal environment [20,21,22,23,24]. Thus, the present work is aimed at the application of the already-documented smart delivery strategy involving the acidity-triggered dissolution [10,11,12,13,14,15,16,17,25,26,27,28] of the metal complex-based ENs.

Au(I) complexes with phosphine derivatives have been already well recognized as a promising basis for anticancer therapeutic agents due to the cascade of chemical transformations triggered by their interactions with biothiols and the enzymes responsible for the intracellular redox balance [29,30,31,32,33]. The Au(I) complexes combining positive charging with enough hydrophobicity are of particular importance from the viewpoint of cytotoxicity due to their potential for mitochondrial localization [29,34]. However, it is well-known that both the entrance and cytotoxicity mechanisms of molecular complexes significantly differ from those of their nanoparticulate forms. Moreover, it has already been demonstrated that the cytotoxicity of [Au_2_L_2_]^2+^ (L is a cyclic PNNP ligand) complexes can be significantly modified through their incorporation into heterometallic colloids based on the counter-ion binding with the anionic hexamolybdenum cluster complexes [35]. It is worth assuming that a similar strategy can be applied in the development of ENs with the controllable release of [Au_2_L_2_]^2+^ triggered by the acidic lysosomal environment.

The previous reports highlight the hexarhenium chalcohydroxo cluster complex [{Re_6_Q_8_}(OH)_6_]^4−^ (Q = S^2−^ or Se^2−^) as the pH-sensitive luminophore due to the pH-driven protonation/deprotonation of the apical ligands [36]. Moreover, the anionic cluster units [{Re_6_Q_8_}(OH)_6_]^4−^ (Re_6_-Q) are able to assemble with [Au_2_L_2_]^2+^ (Au_2_) in aqueous solutions, resulting in the formation of heterometallic colloids exhibiting pH-induced transformations [37].

Thus, the present work introduces [{Re_6_Q_8_}(OH)_6_]^4−^ (Q = S^2−^ or Se^2−^) as the anionic pH-dependent blocks for self-assembly with Au_2_ complexes at pH above 8.0 and disassembly at pH below 5.0. The electrostatically driven pH-dependent assembly/disassembly of Au_2_ complexes with Re_6_-S or Re_6_-Se cluster units is introduced as a proof-of-concept of the rational design of a heterometallic EN able to undergo disassembly in the specific pH conditions of lysosomal compartments. The modes of surface decoration of the as-engineered heterometallic colloids and their cytotoxicity towards cancer and normal cells in correlation with the intracellular trafficking through the lysosomal pathway of the colloids are also discussed in the present work.

## 2. Materials and Methods

### 2.1. Reagents and Materials

Commercial chemicals: Polyethylenimine (PEI branched, MM_averaged_ = 25,000 Da) was purchased from Aldrich Chemistry (Burlington, MA, USA) and lysozyme was purchased from AppliChem (Darmstadt, Germany). Distilled water was used as a solvent. Tris-(hydroxymethyl)-aminomethane (TRIS) extra pure (pH = 8.8) from Scharlau, acetic-acetate (pH = 4.0–5.5), phosphate (pH = 6.0–7.4), and sodium tetraborate (Na_2_B_4_O_7_·10 H_2_O) (pH = 10.1) buffers were used as the buffers.

The salts K_4_[{Re_6_S_8_}(OH)_6_]·8 H_2_O and K_4_[{Re_6_Se_8_}(OH)_6_]·8 H_2_O were synthesized and purified in accordance with previously published procedures [38].

Complex [Au_2_L_2_]Cl_2_ was synthesized by the reaction of 1,5-bis(p-tolyl)−3,7-bis(pyridine-2-yl)−1,5-diaza-3,7-diphosphacyclooctane (PNNP ligand) with gold (I) tetrahydrothiophene (tht) chloride [(tht)AuCl] in 1:1 ligand-to-metal molar ratio [37]. The PNNP ligand was synthesized through a previously published procedure [39].

The aqueous colloids Au_2_Re_6_-S and Au_2_Re_6_-Se were synthesized by the drop-wise addition of 0.3 mL of the aqueous [Au_2_L_2_]Cl_2_ solution (C = 0.6 mM) to 2.7 mL (C = 0.033 mM) of aqueous K_4_[{Re_6_S_8_}(OH)_6_]·8H_2_O (pH = 8.8 adjusted by 0.1 M TRIS) and K_4_[{Re_6_Se_8_}(OH)_6_]·8H_2_O (pH = 10.1 adjusted by 0.02 M borate buffer) solutions, correspondingly, under vigorous stirring. The pH values of the synthetic solutions were adjusted to 8.8 for Re_6_-S, while higher pH (10.1) was applied for Re_6_-Se to control the negative charge of the cluster units for their assembly with Au_2_ through electrostatic attraction with the simultaneous monitoring of the stability of the Au_2_ complex at the applied pH values (Figure S1).The turbid solutions were then subjected to ultrasonic treatment within 20 min at room temperature with the subsequent separation of colloids through centrifugation (15,000 rpm for 30 min at 5 °C). The ultrasonication-centrifugation procedure was repeated twice in order to remove the excess initial components.

The loss of the Re_6_-S and Re_6_-Se clusters during the synthesis, determined by the luminescence data, is 27% and 26%, respectively.

### 2.2. Methods

#### 2.2.1. Dynamic Light Scattering

Dynamic light scattering (DLS) and electrokinetic potential experiments were performed on a Zetasizer Nano instrument (Malvern Instruments, Malvern, UK). Electrokinetic potential values were calculated by the Smoluchowski–Helmholtz equation [40]. Experimental autocorrelation functions were analyzed with the Malvern DTS software (v1.41, Malvern, UK) and the second-order cumulant expansion methods. The average error was ca. 4%. All samples were prepared in deionized water filtered through a PVDF membrane with a Syringe Filter (0.45 µm). All measurements were performed at least in triplicate at 25 °C.

#### 2.2.2. UV-Vis Absorption Spectra

UV-VIS spectra were recorded on Specord 50 Plus (Analytik Jena AG, Jena, Germany) spectrophotometer in 10 mm quartz cuvettes.

#### 2.2.3. Fluorescence Spectroscopy

The emission spectra were recorded on a fluorescence spectrophotometer Hitachi F-7100 (Tokyo, Japan) with stigmatic concave diffraction grating. The excitation of samples was performed at 400 nm and emission was detected at 500–750 nm.

#### 2.2.4. IR Spectroscopy

The infrared spectra were recorded on a Tensor 27 Fourier-transform spectrometer Bruker (Ettlingen, Germany) in a range of 4000–400 cm^−1^ with an optical resolution of 4 cm^−1^ and an accumulation of 32 scans using KBr pressed pellets.

#### 2.2.5. ICP-OES

Re, Au, and P ion concentrations in colloids were identified using a simultaneous inductively coupled plasma optical emission spectrometer (ICP-OES), model iCAP 6300 DUO by Varian Thermo Scientific Company, equipped with a CID detector (168 Third Avenue, Waltham, MA, USA). Together, the radial and axial view configurations enable optimal peak height measurements with suppressed spectral noises. The concentrations of Re, Au, and P ions were determined, respectively, by the spectral lines: 221.426, 242.795, and 178.284 nm. Sc standard was used as the internal standard (10 ppm in each sample) and standards of Re, Au, and P as calibration standards (five-point calibration).

#### 2.2.6. TEM Measurements

Samples were prepared as follows: a drop of 6 μL was taken from the middle of a freshly prepared solution using a dispenser (Biohit Proline Plus, Göttingen, Germany) and applied to a 300 mesh copper grid with a carbon-formvar support film (Agar Scientific, Essex, UK). A drop completely covers the grid. The sample preparation process was carried out at room temperature. Next, the sample was dried in a muffle furnace at 80 °C. TEM images were obtained on a Hitachi HT7700 transmission electron microscope (Tokyo, Japan) at an accelerating voltage of 100 kV (direct observation state).

#### 2.2.7. Powder X-ray Diffraction (PXRD)

PXRD measurements were performed on an automatic Bruker D8 Advance diffractometer equipped with a Vario attachment and Vantec linear PSD using Cu radiation (40 kV, 40 mA) monochromated by a curved Johansson monochromator (λ CuKα1 1.5406 Å) (Bruker Optik GmbH, Ettlingen, Germany). Room temperature data were collected in the reflection mode with a flat-plate sample.

A colloidal solution in water was applied to a silicon plate for the study. To increase the total amount of the sample, several more layers were applied on top of the first one after it dried. Patterns were recorded in the 2θ range between 3° and 90° in 0.008° steps with a step time of 1s. The samples were spun (15 rpm) throughout the data collection. Processing of the obtained data was performed using EVA [41] and TOPAS [42] software packages.

#### 2.2.8. Confocal Laser Microscopy

M-HeLa cells in the amount of 1 × 10^6^ cells/well at a final volume of 2 mL were sown in 6-well plates (Eppendorf AG, Hamburg, Germany) in DMEM (PanEco, St. Petersburg, Russia) supplemented with 1% L-glutamine, 0.1% gentamicin, and 10% FBS (Gibco, Life Technologies, Eugene, OR, USA). After 24-h incubation in a CO_2_ incubator (37 °C, 5% CO_2_) LSZ-Au_2_Re_6_-S and LSZ-Au_2_Re_6_-Se colloids at a final concentration of 1 and 3 µM, respectively, were added to the wells and incubated for 24 h. Untreated M-HeLa cells were used as a negative control.

For lysosomes imaging, M-HeLa cells after incubation with colloids were washed twice with PBS and then the cells were incubated for 30 min with LysoTracker Blue DND 22 (1 μM; Invitrogen, Life Technologies Corp., Eugene, OR, USA). Cells were imaged using a Laser Scanning Confocal Microscope Leica TCS SP5 MP (Leica Microsystems, Wetzlar, Germany). Colloids were excited at 405 nm and the fluorescence emission was collected from 620 to 670 nm. LysoTracker Blue DND 22 was excited at 373 nm and the fluorescence emission was collected from 421 nm to 527 nm. Image analysis was performed in LAS AF software (version 2.2.1 Build 4842, SP5, Leica Microsystems CMS GmbH, Mannheim, Germany) with a colocalization tool. Pearson’s correlation coefficient was used to quantify the correlation between the fluorescence intensities of the LysoTracker Blue DND 22 and colloids.

The lysosomal disruption study was investigated using AO as the indicator. After incubation with NPs, M-HeLa cells were stained with AO (5 μM) for 5 min, followed by three washes with PBS. Finally, the cells were imaged by CLSM (Leica TCS SP5 MP (Leica Microsystems, Wetzlar, Germany)). The excitation of AO was 488 nm, and the emission was 525 nm for green and 625 nm for red.

#### 2.2.9. Statistical Analyses

Pearson’s correlation coefficient was calculated for each captured field of vision (*n* = 50), and at least 50 cells were taken into account for each field. Data are expressed as means ± SE. The significance of differences in normally distributed data was analyzed with Student’s t-test. Differences were regarded to be statistically significant at *p* < 0.05.

#### 2.2.10. Cytotoxicity Assay

The cytotoxic effects of the colloids on human cancer and normal cells were assessed by means of the multifunctional Cytell Cell Imaging system (GE Health Care Life Science, Uppsala, Sweden) using the Cell Viability Bio App, which precisely counts cell numbers and evaluates their viability based on the fluorescence intensity of the dyes 4’,6-diamidine-2-phenylindole (DAPI) and propidium iodide (PI) [43]. Intercalating fluorochrome DAPI penetrates intact membranes of living cells and stains nuclei blue. The high molecular weight dye PI passes only into dead cells with altered membranes, staining them orange. Human clone M-HeLa 11, epithelioid carcinoma of the cervix, strain HeLa, clone M-HeLa from the Collection of type cultures of the Institute of Cytology of the Russian Academy of Sciences, and the Chang liver cell line (human liver cells) by N. F. Gamalei microbiology were used in experiments. The cells were cultured in a standard Eagle’s nutrient medium (PanEco company, Moscow, Russia), supplemented with 10% fetal calf serum and 1% nonessential amino acids. The cells at a concentration of 100,000 cells/mL were plated into a 96-well plate (Eppendorf) (150 μL of medium per well) and incubated under CO_2_ at 37 °C. After incubation for 24 h, the colloids were added at different concentrations (150 μL to each well). The dilutions of the colloids were prepared immediately in nutrient media. The experiments were repeated three times. The untreated M-HeLa and Chang Liver cells were used as controls.

The IC_50_ values were calculated using the online calculator MLA-Quest Graph™ IC50 Calculator (AAT Bioquest, Inc., Sunnyvale, CA, USA) (Version 2021) [44]. The values calculated from the triplicate measurements were averaged.

#### 2.2.11. Cellular Uptake Study

M-HeLa cells (1 × 10^5^ cells/well) at a final volume of 500 µL were sown in a 24-well plate (Eppendorf, Tokyo, Japan). After 24-hour incubation, 1 μM of testing compounds were added to the wells and incubated for 24 h under CO_2_. Cellular uptake was analyzed by flow cytometry (Guava easy Cyte 8HT, MERCK, Kenilworth, NJ, USA). Studies were performed with irradiation by laser V 405 nm, filter 583/26. Untreated cells were used as a negative control. A detailed description of the procedure is introduced in [45]. The studies were carried out in triplicate.

#### 2.2.12. Cell Apoptosis Analysis

M-HeLa cells (1 × 10^6^ cells/well) at a final volume of 2 mL were sown in a 6-well plate. After 24-hour incubation, testing compounds were added to wells. The cells were harvested at 2000 rpm for 5 min and then washed twice with ice-cold PBS (4 °C), followed by resuspension in binding buffer 100 μL. Next, the samples were incubated with 0.35 μL of Annexin V-Alexa Fluor 647 and 0.1 μL of PI for 40 min at room temperature in the dark. Finally, the cells were analyzed by flow cytometry (Guava easy Cyte, MERCK, Kenilworth, NJ, USA). Untreated cells were used as control. A detailed description of the procedure is introduced in [46]. The 20,000 events have been analyzed in the apoptotic assay. The studies were carried out in triplicate.

## 3. Results and Discussion

### 3.1. Synthesis and Characterization of Au_2_Re_6_-Q Colloids

The presentation of the results on the assembly of Au_2_- and Re_6_-Q-blocks should be preceded by a discussion of the pH-dependent transformations of the latter units. The pH-triggered step-wise protonation/deprotonation of the apical OH^–^/H_2_O ligands through the equilibrium (1) is followed by the changing of the cluster’s charge from “–4” in highly alkaline conditions, coming through “–2” in weak alkaline to “0” in neutral with further recharging up to “+2” in acidic solutions [36]. The aforesaid charge transformation affects the water solubility of the cluster forms. In particular, the lowest water solubility is observed for the neutral [{Re_6_Q_8_}(H_2_O)_4_(OH)_2_] form. The nature of Q (Q = S^2−^ or Se^2−^) is another factor in the shifting of the equilibrium (1) [36]. In particular, the equilibrium (1) is shifted to the right in going from Q = S^2−^ to Se^2−^ due to the lower water solubility of [{Re_6_Se_8_}(H_2_O)_4_(OH)_2_] in comparison with [{Re_6_S_8_}(H_2_O)_4_(OH)_2_] [36].

[{Re_6_Q_8_}(OH)_6_]^4^^−^ + *n*H^+^ ↔ [{Re_6_Q_8_}(H_2_O)*_n_*(OH)_6−*n*_]^*n*^^−4^ (1)

The as-prepared colloids were separated from the supernatants by centrifugation to wash out the residual Re_6_-Q and Au_2_. The extraction of both Re_6_-Q and Au_2_ components from the aqueous to the colloidal phase is evident from the spectral analysis of the supernatants (Figure S2). The content of the colloids evaluated by the ICP-EOS method is close to 6:2:4 (Re:Au:P), while the deviations from the ratio being 6:1.6:3.4 for Re_6_-S and 6:2.3:4.0 for Re_6_-Se can be explained by the admixture of residual components. Nevertheless, herein and further the colloids will be designated as Au_2_Re_6_-S and Au_2_Re_6_-Se.

**Figure 1:**
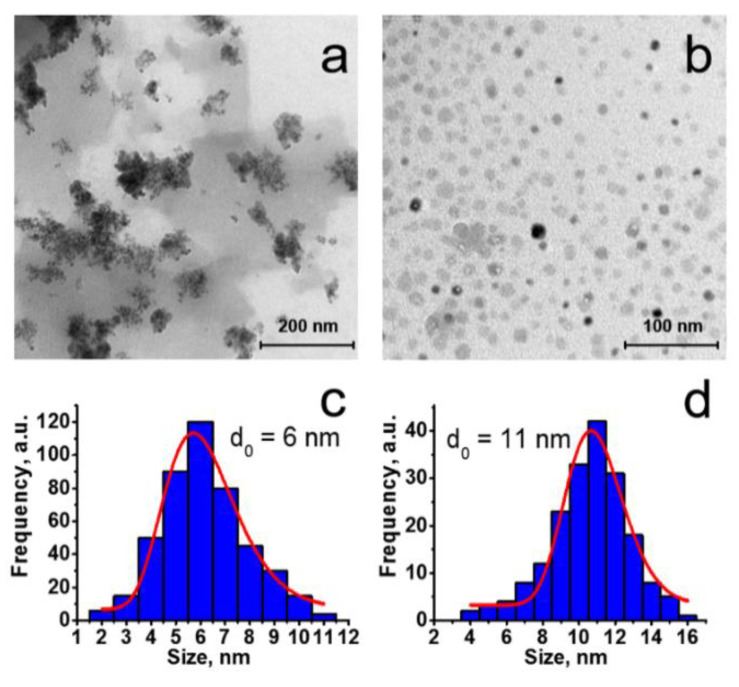
TEM images (**a,b**) and size distribution histograms (**c,d**) of dried samples of Au_2_Re_6_-S (**a,c**) and Au_2_Re_6_-Se (**b,d**) colloids.

The comparative analysis of the IR spectra of the dried Au_2_Re_6_-Q colloids, [Au_2_L_2_]Cl_2_, K_4_[{Re_6_S_8_}(OH)_6_]·8 H_2_O, and K_4_[{Re_6_Se_8_}(OH)_6_]·8 H_2_O indicates the presence of the bands peculiar for the initial [Au_2_L_2_]Cl_2_ complex with broad additional bands of Re_6_-Q clusters (Figure S3). The dried Au_2_Re_6_-Q colloids are manifested in the TEM images (Figure 1a,b) by the aggregates of ultra-small nanoparticles (NPs) with sizes of about 6 and 11 nm, respectively, as is evident from the size distribution histograms (Figure 1c,d), which were made on the basis of the higher-resolution TEM images of the colloids (Figure S4). The aggregation in the dried state is more pronounced for the smaller-sized Au_2_Re_6_-S colloids (Figure 1a).

The PXRD patterns of the dried Au_2_Re_6_-Q colloids (Figure S4) reveal the amorphous nature of the colloids at Q = S^2−^, while the crystallinity extent is greater for the colloids at Q = Se^2−^. This correlates with the nanoparticle size, which is greater in the case of selenide than sulfide counterparts (Figure 1c,d).

The DLS measurements indicate both high polydispersity and the formation of the large-sized aggregates of Au_2_Re_6_-Q colloids in the aqueous and buffered solutions (Table 1). The electrokinetic potentials (**ζ**) of Au_2_Re_6_-Q colloids are far above –30 mV, which agrees well with their high aggregation in the aqueous solutions (Table 1). However, the hydrophobic nature of the Au_2_ block at the interface of the Au_2_Re_6_-Q colloidal nanoparticles should be noted as one more reason for their enhanced aggregation. 

### 3.2. pH-Dependent Leaching of Au_2_ and Re_6_-Q Units

**Figure 2:**
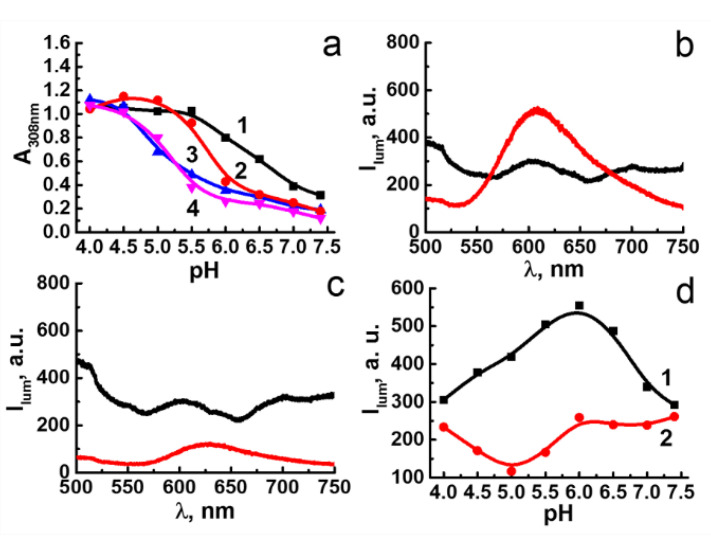
(**a**) Absorbance-pH plots for supernatant solutions of Au_2_Re_6_-S (1), Au_2_Re_6_-Se (2), LSZ-Au_2_Re_6_-S (3), and LSZ-Au_2_Re_6_-Se (4) colloids. (**b,c**) Luminescence of Re_6_-cluster unit in Au_2_Re_6_-S (b) and Au_2_Re_6_-Se (c) at pH = 7.4 (black lines), pH = 5.5 (b, red line), or pH = 5 (c, red line). C_colloids_= 22 μM. (**d**) Luminescence-pH plots of Au_2_Re_6_-S (1) and Au_2_Re_6_-Se (2) colloids at λ_max_ = 607 nm (1) and λ_max_ = 630 nm (2).

As it was previously reported, the Au_2_ complex exhibits high cytotoxicity [35], much higher than that reported for the Re_6_ units [47]. Thus, the extent of the Au_2_ complex leached from Au_2_Re_6_-Q colloids is of great impact on the cytotoxicity of the colloids. The pH-dependent leaching of Au_2_ from Au_2_Re_6_-Q NPs was monitored through the redispersion of the as-synthesized colloids in the buffer solutions at various pHs with the further centrifugation-facilitated phase separation and spectral monitoring of the supernatants (Figure 2). The latter is based on the electronic absorption of Au_2_ in aqueous solutions (Figure S1) manifested by two bands at 250 and 308 nm, where the former interferes with the electronic absorption spectra of Re_6_-Q. Thus, the absorbance values (A) at 308 nm of the supernatant solutions evaluated at different pHs (Figure 2a) allow the quantitative monitoring of the leached Au_2_ complex. The analysis of the A-values plotted vs. pH reveals the disassembly of Au_2_Re_6_-S followed by the leaching of Au_2_ at pH below 7.4, while the disassembly of Au_2_Re_6_-Se requires more acidic conditions (pH < 6.0) (Figure 2a). The enhanced disassembly of Au_2_Re_6_-S vs. Au_2_Re_6_-Se at pH range 6.0–7.4 (Figure 2a) correlates with the higher water solubility of Re_6_-S vs. Re_6_-Se [36]. The extent of the leached Au_2_ complex comes to the same level for Au_2_Re_6_-Q at pH below 5.5, which correlates with the evidence by a naked-eye conversion of Au_2_Re_6_-Q colloids into the true solutions (Figure S5). The disassembly of the colloids can be represented by the equilibrium (2):

[Au_2_L_2_][{Re_6_Q_8_}(H_2_O)_2_(OH)_4_] + 2H^+^ = [{Re_6_Q_8_}(H_2_O)_4_(OH)_2_] + [Au_2_L_2_]^2^ (2)

The red cluster-centered luminescence of Au_2_Re_6_-S is rather low at pHs 6.5–7.4 (Figure 2b,d), while the appearance at pH = 5.5 of the luminescence band peculiar for the “free” Re_6_-S cluster units (Figure 2b) confirm the disassembly of the colloids. The luminescence intensity of Au_2_Re_6_-Se remains unchanged in the wider pH range (Figure 2c,d), being followed by the appearance of the lower intensity luminescence band at pH = 5.0 (Figure 2c). The luminescence intensity (I) of Re_6_-Se units plotted vs. pH (Figure 2d) follows a similar trend as the leaching of Au_2_ (Figure 2a). However, unlike the disassembly of Au_2_Re_6_-S resulting in the high luminescence of the released cluster form [{Re_6_S_8_}(H_2_O)_4_(OH)_2_], the disassembly of Au_2_Re_6_-Se results in poor luminescence at pH = 5.0. This agrees well with the previously reported water solubility of [{Re_6_S_8_}(H_2_O)_4_(OH)_2_] and the insignificant solubility of [{Re_6_Se_8_}(H_2_O)_4_(OH)_2_] [36]. The increase in the luminescence intensity of Au_2_Re_6_-S under acidification from pH 7.4 to 6.0 correlates with the increased leaching of the Au_2_ complex (Figure 2a,d). The luminescence decrease in Re_6_-S at pH below 5.5 derives from the pH-dependent spectral behavior of the cluster itself [36]. Indeed, the profiles of I plotted vs. pH measured for Au_2_Re_6_-Q colloids (Figure 2d) agree with the similar profiles reported for the “free” Re_6_-Q cluster units at similar pHs [36].

### 3.3. PEI-Induced Disassembly of Au_2_Re_6_-Q Colloids and Their Surface Decoration by LSZ

**Figure 3:**
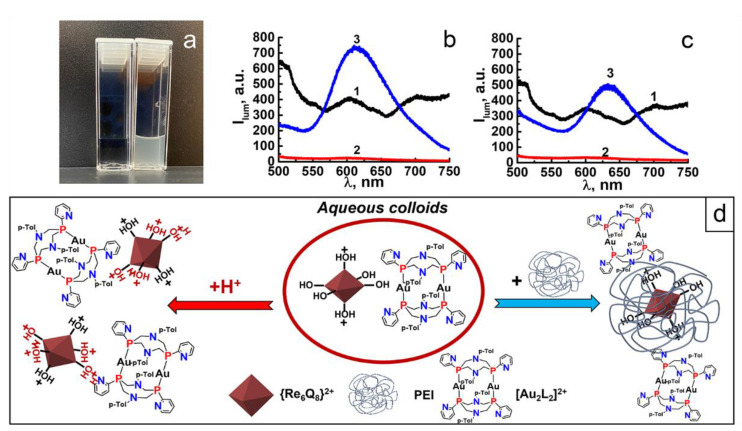
(**a**) Photos of Au_2_Re_6_-Q colloids before (right) and after (left) the addition of PEI. (**b,c**) Luminescence data for Au_2_Re_6_-S (**b**) and Au_2_Re_6_-Se (**c**): 1–colloids; 2–corresponding colloids after treatment by PEI; 3–supernatants. (**d**) Schematic representation of Au_2_Re_6_-Q colloids dissolution upon acidification and treatment by PEI.

It is worth considering the surface decoration of NPs as the factor influencing their disassembly. The negative ζ–values of Au_2_Re_6_-Q colloids (Table 1) derived from their loading with the Re_6_-Q units can be a reason for their interaction with polycationic polyelectrolytes, such as polyethylenimine (PEI) [48]. However, the mixing of the preliminary separated through centrifugation colloids with the solution of PEI (1g∙L^−1^) results in the whole disassembly of Au_2_Re_6_-Q colloid species, as illustrated in Figure 3a. The spectral analysis of the supernatants after the phase separation reveals high luminescence intensity (Figure 3b,c) derived from the Re_6_-Q cluster units wrapped by PEI chains, as schematically demonstrated by Figure 3d [47,48].

The effect of PEI on Au_2_Re_6_-Q colloids prompts searching for another mode of surface decoration. The surface-exposed amino groups of LSZ are a prerequisite for its efficient binding with the inorganic or composite NPs, which, in turn, can affect the interfacial destruction of the NPs [49,50]. The role of LSZ on the acidification-induced disassembly of Au_2_Re_6_-Q colloids is revealed through the spectrophotometric analysis of the supernatants at various pHs (Figure 2a). In accordance with a previous report [50], the concentration level of LSZ required to bind with the colloidal species is about 1.6 µM. The results indicate that the addition of LSZ (1.6 µM) to Au_2_Re_6_-Q colloids affects the pH-dependent leaching of Au_2_, which becomes insignificant within a pH range of 6.0–7.4 for the colloids (Figure 2a). It is worth assuming that the interfacial deposition of LSZ onto Au_2_Re_6_-Q NPs restricts the protonation of the NPs; thus, the disassembly of LSZ-Au_2_Re_6_-Q requires greater acidification than their untreated analogs.

### 3.4. Cytotoxicity and Cell Internalization of Au_2_Re_6_-Q and LSZ-Au_2_Re_6_-Q Colloids

The red luminescence of Au_2_Re_6_-Q and their LSZ-treated analogs is prerequisite for the visualization of the NP cellular uptake behavior. The imaging experiments were preceded by cytotoxicity measurements. The IC_50_ values calculated from the cell viability data of M-HeLa and Chang Liver cell lines incubated by Au_2_Re_6_-Q and LSZ-Au_2_Re_6_-Q (Figure S6) are collected in Table 2.

The presentation of the results on the cell internalization and intracellular localization of LSZ-Au_2_Re_6_-Q colloids is worth preceding by notification of the factors such as size, aggregation, surface charge, and protein corona influencing the endocytic pathway of the nanoparticles [51,52,53]. The large aggregates of Au_2_Re_6_-Q colloids in the buffered solutions amplify their cell internalization through micropinocytosis rather than receptor-mediated endocytosis [54], although the polydispersity of the aggregates (Table 1) indicates the great diversity in their size. Thus, some of the NPs can be internalized through another endocytic mechanism. However, the flow cytometry measurements at the colloids’ concentration below IC_50_ do not visualize the cell internalization of Au_2_Re_6_-Q and their LSZ-treated analogs, since the fluorescence intensity values measured for the incubated cells differ from the negative control value by no more than 5%, which is within the standard error of the method (Figure S7). This can be explained by the low luminescence intensity of the colloids at these concentration conditions. Nevertheless, the IC_50_ values represented in Table 2 indicate the rather high cytotoxic effect of the colloids, which argues for their efficient cell internalization. Moreover, the IC_50_-values of Au_2_Re_6_-Q are on the same level or even smaller than those of [Au_2_L_2_]Cl_2_ (Table 2), while their incorporation into the heterometallic colloids must be the reason for the lower cytotoxicity, as previously reported [35]. This argues for the disassembly of the colloids within the cells, which, in turn, requires the lysosomal pathway of the colloids.

Although the cell viability of both cancer (M-HeLa) and normal (Chang Liver) cell lines is suppressed after incubation with Au_2_Re_6_-Q and LSZ-Au_2_Re_6_-Q colloids (Table 2, Figure S6), the cytotoxic effect of the colloids on the normal cells is detectably decreased after their treatment by LSZ (Table 2). Thus, the LSZ-treated colloids are chosen for the further evaluation of their intracellular pathways. 

**Figure 4:**
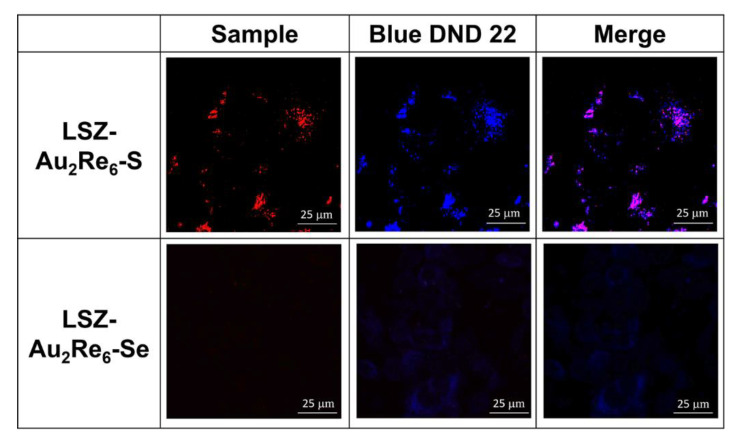
Colocalization analysis of LSZ-Au_2_Re_6_-Q and LysoTracker Blue DND 22 after 24 h of M-HeLa cells incubation.

The specificity of the confocal microscopy technique provides a greater opportunity to visualize the endocytic pathway of low-emitting nanomaterial. The cell internalization and intracellular localization of LSZ-Au_2_Re_6_-S colloids in the M-HeLa cells have been successfully visualized through the red luminescence of the Re_6_-S units by means of the confocal microscopy technique, focusing on their lysosomal localization. The latter has been determined through the staining of the cells incubated with the colloids with LysoTracker Blue DND 22, followed by the quantitative evaluation of the colocalization of the red-emitting NPs and blue emitting LysoTracker within lysosomes by the calculation of Pearson’s correlation coefficient (PCC) [15]. The confocal imaging of the cells incubated with the colloids indicates a high contrasting effect of LSZ-Au_2_Re_6_-S colloids, but a poor one in the case of LSZ-Au_2_Re_6_-Se colloids (Figure 4). In turn, the high contrasting effect of LSZ-Au_2_Re_6_-S allows the evaluation of the PCC value, which is 0.79 ± 0.01 (Figure 4). The high (above 0.5) PCC value of LSZ-Au_2_Re_6_-S colloids indicates their lysosomal localization [15], while the latter is a prerequisite for disassembly in the acidic environment followed by the release of the highly luminescent Re_6_-S units. In this connection, it is worth noting that LSZ-Au_2_Re_6_-Q colloids exhibit rather low luminescence at neutral pH, while the acidification to pH = 5.0 results in the different luminescence response of the colloids arising from the release of bright-emitting Re_6_-S and low-emitting Re_6_-Se cluster units (Figure 2d). This allows for assuming the lysosomal pathway of both NPs as the reason for the different contrasting effects of LSZ-Au_2_Re_6_-S and LSZ-Au_2_Re_6_-Se colloids.

**Figure 5:**
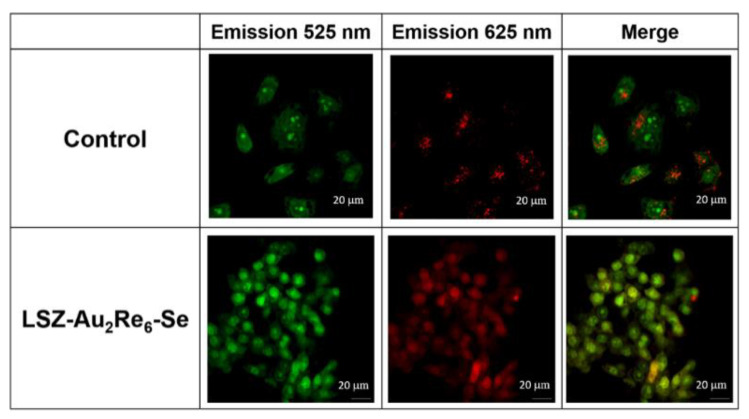
CLSM images showing the rapture of endo/lysosomal membranes in M-HeLa cells incubated by LSZ-Au_2_Re_6_-Se through the AO assay. Cells treated with Acridine Orange only were used as control.

As above-mentioned, the dissolution of the nanomaterial within lysosomes is the prerequisite for lysosome membrane rapture [10,11,12,13,14,15,16,17]. In turn, the cargo-induced rapture of lysosomal compartments can be efficiently monitored through the assay based on Acridine Orange (AO) [22]. The lysosomal localization of AO is followed by the red emission, while its release from the lysosomal compartments into the cytoplasm is visualized by the green emission [22]. The poor contrasting effect of LSZ-Au_2_Re_6_-Se allows for following the rapture of endo/lysosomal membranes in M-HeLa cell samples through the AO assay, while the red emission of LSZ-Au_2_Re_6_-S interferes with the red emission of AO. Figure 5 demonstrates the red spots in the intact cells derived from the localization of AO in the lysosomal compartments, while more blurred green spots derive from the cytosolic distribution of AO. The AO assay reveals the specificity in the localization of the red and green spots in the cells incubated with LSZ-Au_2_Re_6_-Se colloids. The specificity manifested by the blurred red spots interfering with the green ones argues for the enhanced rapture of the cell lysosomes, in turn, derived from the disassembly of LSZ-Au_2_Re_6_-Se colloids. Most likely, the lysosomal localization of LSZ-Au_2_Re_6_-S is also followed by the rapture of the lysosomal membrane, as observed for LSZ-Au_2_Re_6_-Se. Thus, the confocal microscopy results provide both direct evidence of the cell internalization followed by the lysosomal pathway for LSZ-Au_2_Re_6_-S (Figure 4) and indirect evidence of the lysosomal pathway for LSZ-Au_2_Re_6_-Se colloids (Figure 5). This, along with the aforesaid disassembly of LSZ-Au_2_Re_6_-Q colloids in acidified solutions, is the reason for the release of toxic [Au_2_L_2_]^2+^ complex into the cell cytoplasm, which correlates with the high cytotoxicity of the colloids (Table 2).

Indeed, the IC_50_-values of Au_2_Re_6_-Q are on the same level or even smaller than those of [Au_2_L_2_]Cl_2_ (Table 2), while their incorporation into the heterometallic colloids must be the reason for the lower cytotoxicity, as previously reported [35]. Thus, the lysosomal pathway of Au_2_Re_6_-Q colloids and their LSZ-treated counterparts results from a fusing of late endosomes with lysosomes [55], followed by their disassembly in the acidic lysosomal environment, and the release of the toxic [Au_2_L_2_]^2+^ complex into the cell cytoplasm is responsible for the observed high cytotoxicity of the colloids (Table 2). The above-mentioned difference in the leaching extents of Au_2_Re_6_-S and Au_2_Re_6_-Se colloids is of insignificant impact on the IC_50_-values, while the cytotoxicity of the LSZ-treated colloids differs from that of their untreated analogs (Table 2). The IC_50_-values of LSZ-Au_2_Re_6_-Se colloids are detectably higher than those of Au_2_Re_6_-Se, which agrees well with the restricted leaching of the Au_2_ units at pH range 5.0–7.0, while the cytotoxicity of Au_2_Re_6_-S colloids remains unchanged in the case of the Chang Liver cell samples and somewhat increases towards the cancer cells (Table 2). This can be explained by the so-called “protein corona” effect [56,57,58], which influences various aspects of the intracellular behavior of nanoparticles.

**Figure 6:**
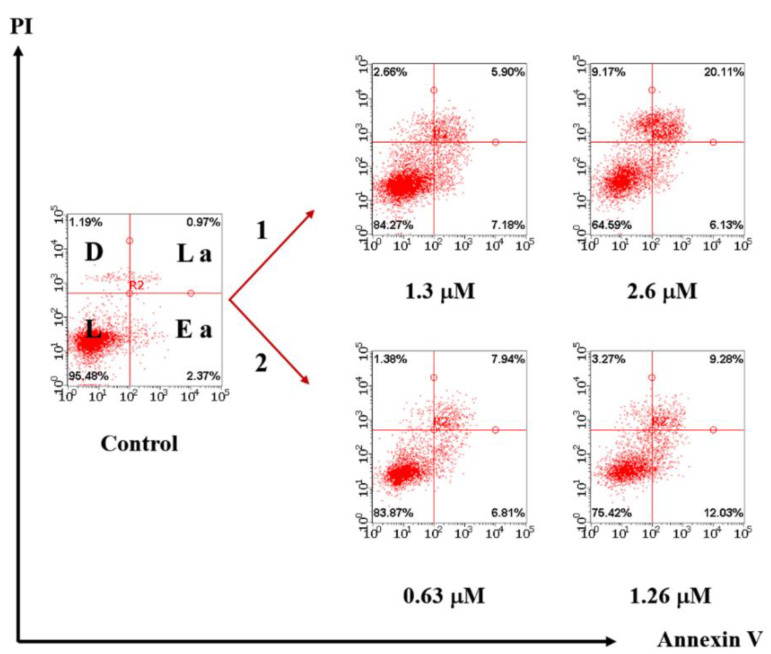
Flow cytometry analysis of M-HeLa cells treated with different concentrations of Au_2_Re_6_-S (1) and LSZ-Au_2_Re_6_-S (2) after Annexin V and PI staining. The values are presented as the mean ± SD (*n* = 3): * *p* < 0.01 vs. the control group. L—living cells; D—dead cells; E. a.—early apoptotic cells; L. a.—late apoptotic cells.

The selective lysosomal localization of the colloids followed by their pH-induced disassembly results in the lysosomes rapturing, which can be a reason for the apoptotic cell death mechanism [11]. The apoptotic assay indicates that the apoptotic pathway detectably contributes to the M-HeLa cells’ death under their incubation by both Au_2_Re_6_-Q colloids and their LSZ-treated counterparts (Figure 6 and Figure S8), while only the necrotic processes are visualized for Au_2_ complexes [35]. The comparative analysis of the apoptotic assay results reveals the greater contribution of the necrotic processes up to 36% in the case of Au_2_Re_6_-Se colloids, although even the aforesaid value is below that reported for the Au_2_ complex [35]. This differentiates Au_2_Re_6_-Q colloids from the Au_2_ complex, although their IC_50_ values are close to each other. Thus, to the best of our knowledge, this work for the first time demonstrates the pH-driven release of both luminescent and toxic metal complex blocks from metal complex-based ENs as the application of the smart delivery strategy that has already been documented for the inorganic nanomaterials [10,11,12,13,14,15,16,17].

## 4. Conclusions

The ability of [{Re_6_Q_8_}(OH)_6_]^4−^ (Q = S^2−^ or Se^2−^) cluster complexes to undergo the protonation/deprotonation of the apical hydroxo/aqua ligands followed by the change of their charge under acidic or basic environment highlights the clusters as promising units for the pH-dependent assembly with [Au_2_L_2_]^2+^ (Au_2_) cationic complex resulting in the development of heterometallic Au_2_Re_6_-Q colloids. The disassembly of Au_2_Re_6_-Q colloids in acidic (pH = 4.5) conditions modeling a lysosomal environment is followed by the release of the red-emitting Re_6_-Q and the cytotoxic Au_2_ units. The highly basic microenvironment of polyethylenimine chains triggers the disassembly of Au_2_Re_6_-Q colloids, while their interaction with the protein LSZ shifts the pH values required for the disassembly of colloids to more acidic conditions. The high luminescence intensity of Re_6_-S units revealed under the disassembly of LSZ-Au_2_Re_6_-S is a prerequisite for the determination of their high lysosomal localization extent through the colocalization assay. In turn, the low luminescence of Re_6_-Se units in the same acidified conditions allows us to reveal the rapture of the lysosomal membrane in the cells incubated with LSZ-Au_2_Re_6_-Se through the use of the Acridine Orange-based assay. The lysosomal pathway of the colloids followed by their endo/lysosomal escape correlates with the cytotoxicity of LSZ-Au_2_Re_6_-Q being on the same level as that of Au_2_ complexes. However, the significant contribution of the apoptotic pathway differentiates the cytotoxic effect of the colloids from that of the Au_2_ complex, which predominantly arises from the necrotic processes. 

## Figures and Tables

**Table 1:** Evaluated through the size distribution by number (*d_num_*) diameter values, polydispersity indices (PDI), and electrokinetic potentials (**ζ**) of Au_2_Re_6_-Q colloids both itself and after the addition of lysozyme (LSZ) (1.6 µM). Concentration of phosphate buffer is 0.01 M.

	***d_num_*, nm**	**PDI**	**ζ, mV**
Au_2_Re_6_-S	498–520	~1	–10.8 ± 0.6
Au_2_Re_6_-Se	400–460	0.846	–17.1 ± 0.8
Phosphate buffer solutions (pH = 7):			
Au_2_Re_6_-S	630–1100	~1	–5.0 ± 0.5
LSZ-Au_2_Re_6_-S	620–1040	~1	–6.0 ± 0.6
Au_2_Re_6_-Se	340–530	0.9	–6.9 ± 0.6
LSZ-Au_2_-Re_6_-Se	460–530	0.9	–4.2 ± 0.7

**Table 2:** IC_50_ values determined for M-HeLa and Chang Liver cell lines after incubation by [Au_2_L_2_]Cl_2_ complex, Au_2_Re_6_-Q, and LSZ-Au_2_Re_6_-Q colloids.

	IC_50_, μM	
	M-HeLa	Chang Liver
[Au_2_L_2_]Cl_2_	2.0 *	3.0 *
Au_2_Re_6_-S	2.24 ± 0.17 **	1.85 ± 0.20 **
LSZ-Au_2_Re_6_-S	1.20 ± 0.10 **	2.59 ± 0.18 **
Au_2_Re_6_-Se	1.70 ± 0.13 **	1.70 ± 0.11 **
LSZ-Au_2_Re_6_-Se	3.40 ± 0.24 **	2.90 ± 0.17 **
LSZ	>30	>30

* Values are taken from the previously published work [35]. ** Refer to the concentration of the Re_6_-Q blocks.

## Data Availability

Not applicable.

## Supplementary Materials

The following supporting information can be downloaded at: https://www.mdpi.com/article/10.3390/nano12183229/s1, Figure S1: (a) UV-Vis spectra of Au_2_ complex at 4 (black), 7 (red) and 10.1 (blue) pH values. C= 0.01 mM. (b) UV-Vis spectra of Re_6_-S (black) and Re_6_-Se (red) clusters at 8.8 and 10.1 pH values, respectively. C= 0.03 mM; Figure S2: (a, b) Luminescence of aqueous solutions of Re_6_-S (a) and Re_6_-Se (b) clusters (C=22 μM, blue lines), Au_2_Re_6_-S (a) and Au_2_Re_6_-Se (b) colloids (C=22 μM, black lines) and supernatants (red lines); Figure S3: Infrared spectra of initial K_4_[{Re_6_S_8_}(OH)_6_] (black) and K_4_[{Re_6_Se_8_}(OH)_6_] (magenta) clusters, Au_2_ complex (red) and dried Au_2_Re_6_-S (blue) and Au_2_Re_6_-Se (green) colloids. The spectra of dried Au_2_Re_6_-S / Au_2_Re_6_-Se colloids represent the sum of spectra of initial K_4_[{Re_6_S_8_}(OH)_6_]/K_4_[{Re_6_Se_8_}(OH)_6_] clusters (1637, 904, 845 and 475 cm^−1^) and Au_2_ complex (1612, 1572, 1514, 1193, 1040 cm^−1^). The higher intensity of band at ~1040 cm^−1^ in the spectrum of Au_2_Re_6_-S (blue) in comparison with initial Au_2_ complex (red) is related to the presence of residual amounts of TRIS buffer used in the synthesis; Figure S4: (a) TEM image of Au_2_Re_6_-S with a magnification of 120K: a fragment of the analyzed area for plotting the particle size distribution. (b) Experimental diffraction patterns from Au_2_Re_6_-S (1) and Au_2_Re_6_-Se (2). The curves are shifted relative to each other along the intensity axis for visual clarity; Figure S5: Photos of pH-dependent dissolution of Au_2_Re_6_-S (left) and Au_2_Re_6_-Se (right) colloids. C_colloids_= 22 μM, C_buffers_= 0.01 M; Figure S6: Viability of M-HeLa (a) and Chang Liver (b) cells incubated with different concentrations of Au_2_Re_6_-S (black lines), LSZ-Au_2_Re_6_-S (red lines), Au_2_Re_6_-Se (blue lines), LSZ-Au_2_Re_6_-Se (magenta lines). (c) Viability of M-HeLa (black line) and Chang Liver (red line) cells incubated with different concentrations of LSZ. The error bars represent standard deviation of the mean values; Figure S7: (a) Cellular uptake study in M-HeLa cells: control (white), Au_2_Re_6_-S (red) and LSZ-Au_2_Re_6_-S (blue) colloids. (b) Cellular uptake study in M-HeLa cells: control (white), Au_2_Re_6_-Se (red) and LSZ-Au_2_Re_6_-Se (blue) colloids. C_colloids_ = 1 μM; Figure S8: Flow cytometry analysis of M-HeLa cells treated with different concentrations of Au_2_Re_6_-Se (1) and LSZ-Au_2_Re_6_-Se (2) after Annexin V and PI staining. The values are presented as the mean ± SD (n = 3): * *p* < 0.01 vs. the control group. L—living cells; D—dead cells; E. a.—early apoptotic cells; L. a.—late apoptotic cells.
