# Supplementary material for: pH-Driven Intracellular Nano-to-Molecular Disassembly of Heterometallic [Au2L2]{Re6Q8} Colloids (L = PNNP Ligand; Q = S2− or Se2−)
Source: Nanomaterials (Basel). 2022 Sep 17;12(18):3229. doi: 10.3390/nano12183229 (PMC9505965; doi:10.3390/nano12183229)
Supplement: Supplementary file 1 [file nanomaterials-12-03229-s001.zip › nanomaterials-1903117-supplementary.pdf]

Supplement File

pH-Driven Intracellular Nano-to-Molecular Disassembly of Heterometallic $[Au_2L_2]\{Re_6Q_8\}$ Colloids (L = PNNP Ligand; Q = S^{2-} or Se^{2-})

Bulat Faizullin [1], Irina Dayanova [1], Igor Strelnik [1], Kirill Kholin [2], Irek Nizameev [1], Aidar Gubaidullin [1], Alexandra Voloshina [1], Tatiana Gerasimova [1], Ilya Kashnik [3], Konstantin Brylev [3], Guzel Sibgatullina [4], Dmitry Samigullin [4,5], Konstantin Petrov [1], Elvira Musina [1], Andrey Karasik [1] and Asiya Mustafina [1,*]

[1] Arbuzov Institute of Organic and Physical Chemistry, FRC Kazan Scientific Center of RAS, 8 Arbuzov Street, 420088 Kazan, Russia
[2] Department of Nanotechnology in Electronics, Kazan National Research Technical University Named after A.N. Tupolev-KAI, 10 K. Marx Street, 420111 Kazan, Russia
[3] Nikolaev Institute of Inorganic Chemistry, SB RAS, 3 Academician Lavrentiev Avenue, 630090 Novosibirsk, Russia
[4] Kazan Institute of Biochemistry and Biophysics, FRC Kazan Scientific Center of RAS, 2/31 Lobachevski Street, 420111 Kazan, Russia
[5] Institute for Radio-Electronics and Telecommunications, Kazan National Research Technical University Named after A.N. Tupolev-KAI, 10 K. Marx Street, 420111 Kazan, Russia
* Correspondence: asiyamust@mail.ru

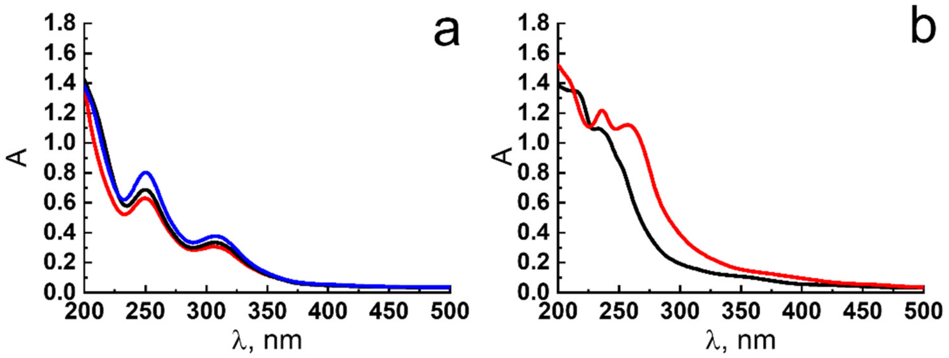

Figure S1. (a) UV-Vis spectra of Au_2 complex at 4 (black), 7 (red) and 10.1 (blue) pH values. C= 0.01 mM. (b) UV-Vis spectra of Re_6-S (black) and Re_6-Se (red) clusters at 8.8 and 10.1 pH values, respectively. C= 0.03 mM.

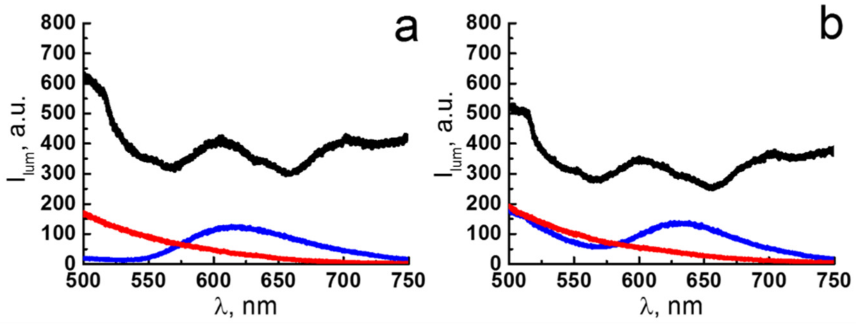

Figure S2. (a, b) Luminescence of aqueous solutions of Re_6-S (a) and Re_6-Se (b) clusters (C=22 µM, blue lines), Au_2Re_6-S (a) and Au_2Re_6-Se (b) colloids (C=22 µM, black lines) and supernatants (red lines).

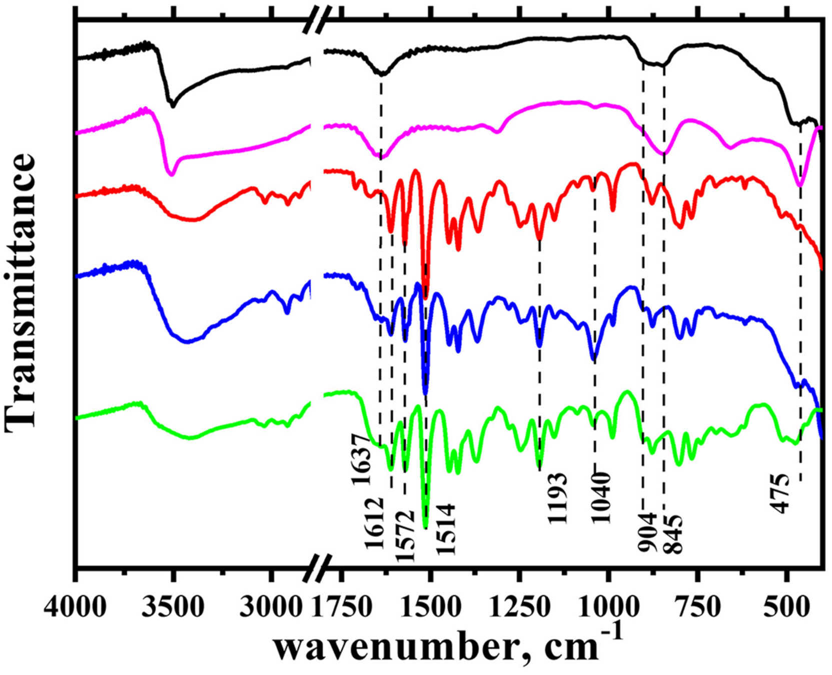

Figure S3. Infrared spectra of initial $K_4[\{Re_6S_8\}(OH)_6]$ (black) and $K_4[\{Re_6Se_8\}(OH)_6]$ (magenta) clusters, Au_2 complex (red) and dried Au_2Re_6-S (blue) and Au_2Re_6-Se (green) colloids. The spectra of dried Au_2Re_6-S / Au_2Re_6-Se colloids represent the sum of spectra of initial $K_4[\{Re_6S_8\}(OH)_6]$ / $K_4[\{Re_6Se_8\}(OH)_6]$ clusters (1637, 904, 845 and 475 cm^{-1}) and Au_2 complex (1612, 1572, 1514, 1193,

1040 cm⁻¹). The higher intensity of band at ~1040 cm⁻¹ in the spectrum of Au_2Re_6-S (blue) in comparison with initial Au_2 complex (red) is related to the presence of residual amounts of TRIS buffer used in the synthesis.

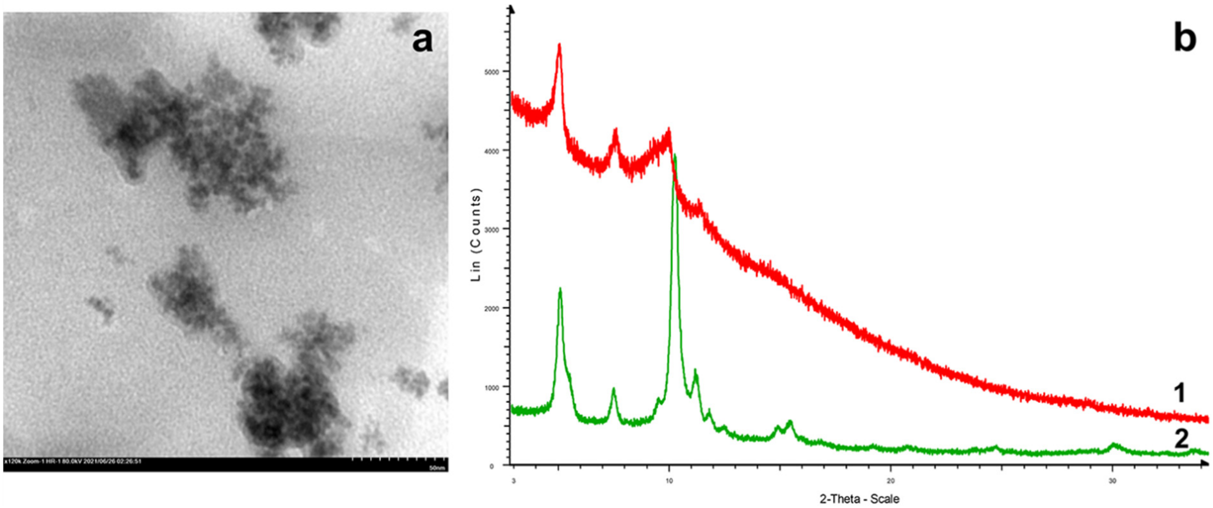

Figure S4. (a) TEM image of Au_2Re_6-S with a magnification of 120K: a fragment of the analyzed area for plotting the particle size distribution. (b) Experimental diffraction patterns from Au_2Re_6-S (1) and Au_2Re_6-Se (2). The curves are shifted relative to each other along the intensity axis for visual clarity.

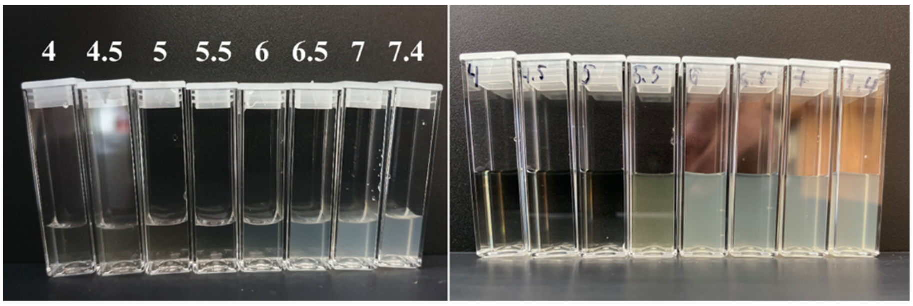

Figure S5. Photos of pH-dependent dissolution of Au_2Re_6-S (left) and Au_2Re_6-Se (right) colloids. $C_{colloids}$= 22 µM, $C_{buffers}$= 0.01 M.

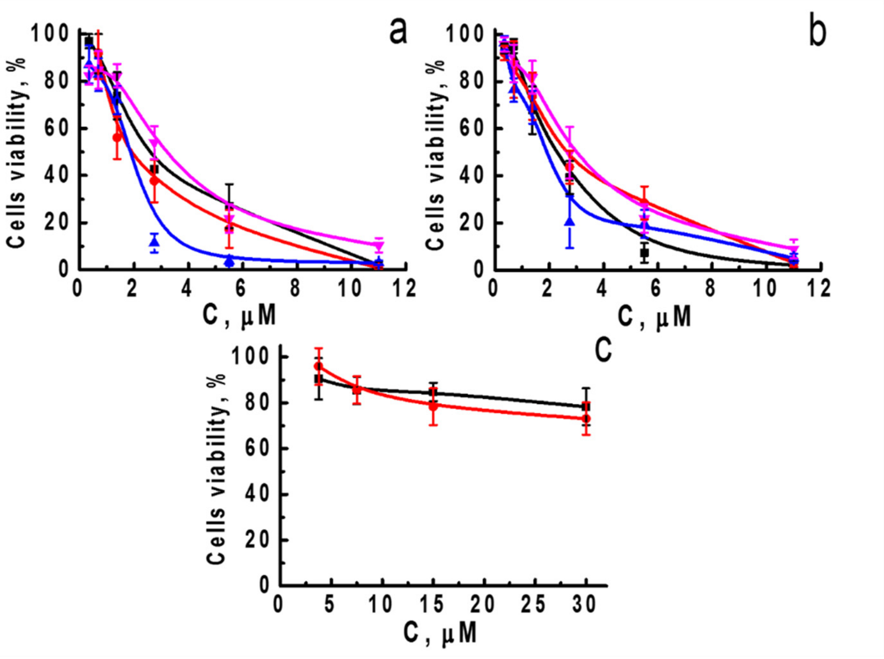

Figure S6. Viability of M-HeLa (a) and Chang Liver (b) cells incubated with different concentrations of Au_2Re_6-S (black lines), LSZ-Au_2Re_6-S (red lines), Au_2Re_6-Se (blue lines), LSZ-Au_2Re_6-Se (magenta lines). (c) Viability of M-HeLa (black line) and Chang Liver (red line) cells incubated with different concentrations of LSZ. The error bars represent standard deviation of the mean values.

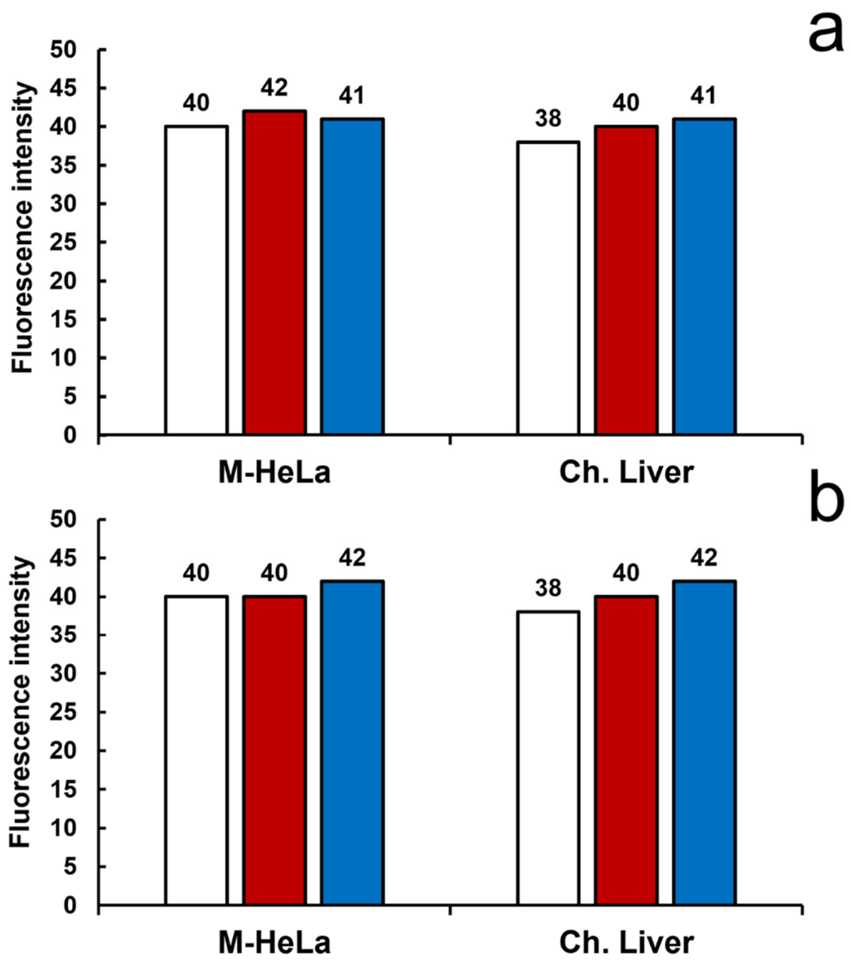

Figure S7. (a) Cellular uptake study in M-HeLa cells: control (white), Au_2Re_6-S (red) and LSZ-Au_2Re_6-S (blue) colloids. (b) Cellular uptake study in M-HeLa cells: control (white), Au_2Re_6-Se (red) and LSZ-Au_2Re_6-Se (blue) colloids. $C_{colloids}$ = 1μM.

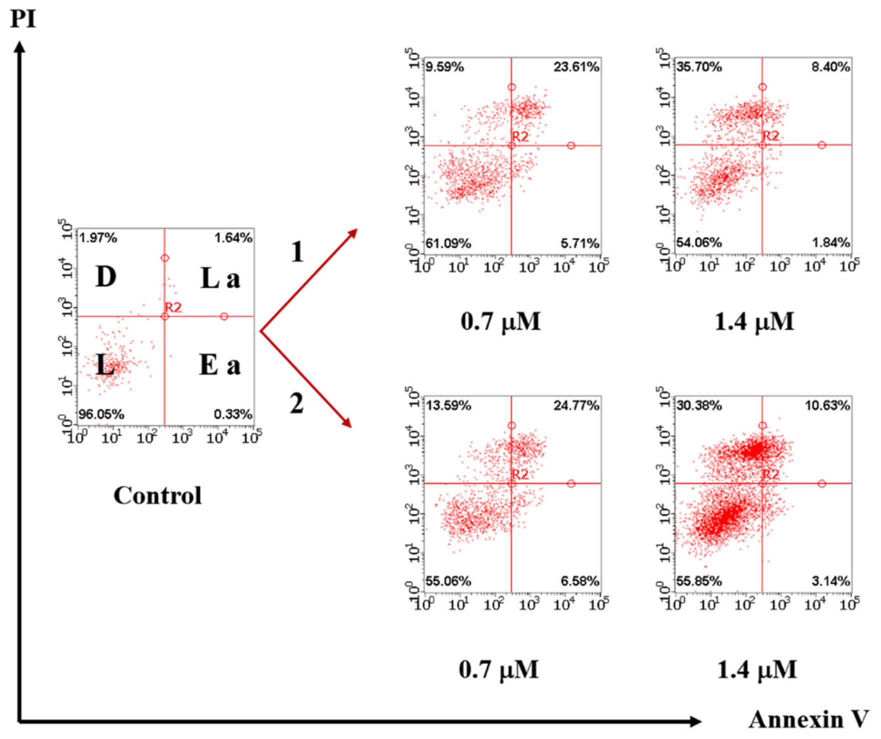

Figure S8. Flow cytometry analysis of M-HeLa cells treated with different concentrations of Au_2Re_6-Se (1) and LSZ-Au_2Re_6-Se (2) after Annexin V and PI staining. The values are presented as the mean ± SD (n = 3): *$p < 0.01$ vs. the control group. L – living cells; D – dead cells; E. a. – early apoptotic cells; L. a. – late apoptotic cells.
